# Molecular identification of *Mycoplasma synoviae* from seroprevalent commercial breeder farms at Chittagong district, Bangladesh

**DOI:** 10.14202/vetworld.2016.1063-1069

**Published:** 2016-10-10

**Authors:** Md. Inkeyas Uddin, Md. Harisul Abid, Md. Shafiqul Islam, Tofazzal Md. Rakib, Ashim Baran Sen, Shah Mohammed Ziqrul Haq Chowdhury, Md. Nurul Anwar, Kazi Md. Kamaruddin

**Affiliations:** 1 Poultry Research and Training Centre, Chittagong Veterinary and Animal Sciences University, Khulshi - 4225, Chittagong, Bangladesh; 2 Department of Livestock Services, People’s Republic of Bangladesh; 3 Department of Pathology and Parasitology, Chittagong Veterinary and Animal Sciences University, Khulshi - 4225, Chittagong, Bangladesh; 4 Livestock Division, Bangladesh Agricultural Research Council, Dhaka - 1202, Bangladesh; 5 Port City International University, Chittagong, Bangladesh

**Keywords:** breeder farm, *Mycoplasma synoviae*, polymerase chain reaction, respiratory infection, risk factors, seroprevalence

## Abstract

**Aim:**

Worldwide, *Mycoplasma synoviae* (MS) is an important pathogen of poultry, especially for chicken and turkey. It causes respiratory tract infection and infectious sinusitis. The study was conducted to determine the seroprevalence of MS infection with associated risk factors and identification of MS organism in unvaccinated flocks of commercial breeder farms of the Chittagong district, Bangladesh.

**Materials and Methods:**

A total of 365 serum samples were collected and tested for MS using serum plate agglutination (SPA) test for determination of MS seroprevalence. On the other hand, tracheal swabs were collected from each seropositive flocks for polymerase chain reaction (PCR) to determine the presence of MS organism.

**Results:**

Among the farms, the highest prevalence was found to be 69% and the lowest prevalence was 28% with the average 60%. The seroprevalence of MS infection in breeder farms was highest 70% with the flock size >10,000 birds, whereas it was lowest 57% in the flocks ranging from 4000 to 7000. According to age group, the prevalence was found highest 70% in >60 weeks age group of birds and lowest 42% in 10-19 weeks group. The seroprevalence of MS in winter season was found as highest as 64%, whereas it was found lowest 60% in the summer season. There was a statistically significant difference (p<0.01) among the seroprevalence of MS in different breeder farms, flock size, and age groups, but there was no significant (p>0.05) difference in the winter, summer, and rainy season. To confirm the presence of MS in the samples, PCR test was applied using specific primers to amplify a 214 bp region of the 16S rRNA gene of the organism. In PCR, all seropositive flocks showed a positive result for MS.

**Conclusion:**

As the plate agglutination test result showed 100% similar with PCR result, it can be suggested that agglutination test is better than molecular and culture techniques for MS detection and it is also cheaper and less time-consuming method.

## Introduction

*Mycoplasmas* are widespread in nature and infect a wide range of hosts. Species from the genus *Mycoplasma* have been isolated (over 110 species) from mammals, birds, reptiles, and fish [1]. Avian mycoplasmosisis caused by several pathogenic *Mycoplasmas* such as *Mycoplasmagallisepticum*, *Mycoplasmasynoviae* (MS), *Mycoplasmameleagridis*, and *Mycoplasmaiowae* [2]. Among them, MG and MS are the most important and are listed as notifiable diseases by the OIE. MS infection is primarily a disease of chicken and turkeys but also infects many other domestic and wild birds worldwide [3,4]. It causessynovitis, chronic respiratory tract disease, and retarded growth in chickens and turkeys [5].

MS can potentially be present in backyard poultry flocks [6,7]. In the breeder flock, the infection causes decreased feed efficiency, poor carcass quality, and suboptimal egg production. As in other poultry producing countries, mycoplasmosis is one of the important disease problems for poultry in Bangladesh, for both commercial and breeder farms [8-10]. In Bangladesh, the prevalence of MS was reported to be 61-67% [11]. Mycoplasmosis may be diagnosed by different methods such as morphology of causal agents, cultural characteristics; physical, biochemical, and serological properties [8]. In general, *Mycoplasma* infections are diagnosed using serological methods and eventually by polymerase chain reaction (PCR) [12]. Various serological tests have been developed for detection of antibodies against MS [13]. The serum plate agglutination (SPA) test could be used as a tool for quick detection of avian *Mycoplasma* infection [14]. Besides, tracheal and cloacal swabs are also used in the identification of the agent using PCR [15]. MS infections are often associated with other diseases, so the use of advanced techniques such as PCR is a tool of great importance in diagnosis of MS infections with achieving greater accuracy, which leads to a better understanding of the pathogen affecting avian populations [16]. Control of pathogenic MS consists of three general approaches: Maintaining flocks free of infection, medication, or vaccination. The most effective method of controlling MS infection is regular monitoring of the flocks and eliminating the positive flocks [17]. Chittagong is considered to be a poultry zone with a number of breeder farms in the area, but less emphasis has been given to diagnose the *Mycoplasma* infection in breeder farm, and no work has been reported on MS in this area. The aim of the study was to determine the prevalence ofMS using SPA and molecular method in the breeder farms of the Chittagong district.

## Materials and Methods

### Ethical approval

Not required for this study.

### Study area and season

The study was conducted in the breeder farms of Chittagong District from January 2012 to December 2012, encompassing summer (March to May), winter (November to February) and rainy (June to October) seasons. The research work was carried out in the Poultry Research and Training Centre (PRTC) Laboratory, Chittagong Veterinary andAnimal Sciences University, Khulshi, Chittagong.

### Sampling

Sample size was determined using the standard formula adopted from Araoye [18], Iloh *et al*. [19] N=z^2^pq/d^2^ Where, N=sample size, z=1.96 confidence interval, p=prevalence, which is 61% for MS [11], d=5% allowable error, q=1−p. Using this formula, the minimum sample size was calculated to be around 365 for the breeder chickens for the Chittagong district in Bangladesh.

A total of 368 blood samples were randomly collected from the breeder farms where the birds were not vaccinated against MS. About 1-1.5 ml of blood was collected from wing vein using a fresh disposable plastic syringe (3 ml volume) for each bird. The blood samples were kept at room temperature for about 1-2 h and then centrifuged at 1500 rpm for 10 min using a bench centrifuge (VELOCITY 18R^™^ refrigerated centrifuge). A clean straw-colored serum was seen up and around the clotted clump that was poured into a labeled Eppendorftube and stored at −20°C until used.

### SPA test

The SPA test was conducted using the method as described in the work of Sarkar *et al*. [20]. In this study, crystal violet stained MS commercial antigen (Bio Vac.) was used. A 25 µl volume of antigen was placed side by side on a glass plate with 25 µl of the serum using a micropipette. The serum and the antigen were then mixed well by stirring with a small toothpick followed by gentle rocking. Results were read within 2 min over a light source. In positive cases, granules were formed slowly which could be seen during rocking. In the negative cases, no such granules were formed. All SPA results were recorded as +, ++, and +++ denoting, respectively, as small, medium, and large to very large clumps. The strength of the agglutination reaction was measured as − = No clumps, no background clearing; + = Small clumps, no background clearing; ++ = Medium sized clumps, almost complete background clearing; +++ = Large clumps, complete background clearing.

### Molecular identification

For PCR test, tracheal swab samples were taken randomly from each of the non-vaccinated seropositive breeder flocks among the studied breeder farms. Swab samples were stored in Phosphate buffered saline (PBS) at 4°C overnight. DNA extraction was than performed according to the protocol adopted by Ogino *et al* [21].

#### DNA extraction

DNA was extracted using the protocol described in brief [21]. Swab samples suspended in 1 ml of PCR-grade PBS in a 1.5 ml Screw-cap Eppendorf tube. The suspension was centrifuged for 30 min at 14,000 g at 4°C, and supernatantwas carefully removed with a Pasteur Pipette. The remaining pellet was suspended in 25 µl PCR-grade water. The tubes with the contents were boiled for 10 min and then placed on ice for 10 min. Then, centrifugation at 14,000 g for 5 min, the supernatant containing the DNA was used as template DNA for PCR.

#### PCR

The reaction mixture was prepared in a separate clean area using a set of dedicated pipettes. For one 50 µl PCR reaction, the mixture containing H_2_O ultrapure 35.75 µl, 10×PCR Buffer 5.00 µl, Dntp (10 Mm) 1.00 µl, forward primer (20 pmole/µl) 0.50 µl, reverse primer (20 pmole/µl) 0.50 µl, taq (5 U/µl) 0.25 µl, and MgCl_2_ (50 Mm) 2.00 µl (Promega^®^). A 45 µl volume of the reaction mixture was dispensed into each PCR tube. The tubes were then taken to another clean area where the appropriate DNA samples (5 µl) were added to each tube. Positive and negative controls were used in each run. The thermal profile consisted 40 cycles of initial denaturation at 94°C for 5 min, denaturing at 94°C for 30 s, annealing at 55°C for 30 s, and extension at 72°C for 1 min, followed by final extension 72°C for 5 min and final storage at 4°C. A negative control did not contain template DNA and consisted of PCR master mix, sets of primers, and nuclease free water. Primer used in this study was previously reported by OIE [13] and Pérez *et al*. [22]. Details of the primers used for PCR are shown in Table-1.

**Table-1:** Details of the primers used for PCR.

Primer name	Gene	Nucleotide sequence (5′-3′)	Product size	Reference
MS-F MS-R	16S r RNA	5´GAG AAG CAA AAT AGT GAT ATC A-3´ 5´CAG TCG TCT CCG AAG TTA ACA A-3´	214 bp	[22]

PCR=Polymerase chain reaction

#### Gelelectrophoresis

PCR product was analyzed by electrophoresis on 1.5% agarose gel, containing SYBR green for 30-40 min at 100v, and examined under ultraviolet (UV) light using a UVlight transilluminator (UV Star, Biometra, Germany).

### Statistical analysis

All data were entered into a spreadsheet program (Excel 2007, Microsoft Corporation) and transferred to Stata 11 (Intercooled Stata 11, Stata Corporation, College Station, Texas, USA) for analysis. The difference in the seroprevalence rate of MS in between variables was shown using Chi-square test.

## Results

To study the seroprevalence of MS, SPA test was done based on the previous research works where the sensitivity and specificity of SPA test were compared with the culture, PCR, and various commercial enzyme-linked immune sorbent assay tests [23-25]. In the present study, sera samples were collected from the different breeder farms of Chittagong district where the birds were not vaccinated against MS. After collection of the samples, SPA test was done followed by PCR as a confirmatory test for identification of the organism as the culturing of MS is costly, time-consuming, and inconclusive [24,26]. Details of the seroprevalenceof MS with different risk factors are shown in Table-2.

**Table-2:** Seroprevalence of *M.synoviae* with different risk factors.

Risk factors	Level of risk factors	Positive percentage				Significance value

		+	++	+++	Total	
Farm	1	19.51	28.05	14.63	62.19	0.002
	2	23.40	25.53	17.02	65.95	
	3	18.37	4.08	6.12	28.57	
	4	35.62	19.18	10.96	65.76	
	5	32.69	21.15	15.38	69.22	
	6	29.63	20.92	13.59	64.14	
Flock size	4000-7000	24.32	20.5	11.49	56.76	0.004
	7001-10,000	33.33	18.18	8.33	59.84	
	>10,000	20.45	25.00	25.00	70.45	
Age	10-19 weeks	25.35	12.68	4.23	42.26	0.000
	20-29 weeks	21.15	15.38	25.00	61.53	
	30-39 weeks	38.89	19.44	4.17	62.50	
	40-49 weeks	22.22	28.89	13.33	64.44	
	50-59 weeks	20.00	31.67	16.67	68.34	
	>60 weeks	27.94	20.59	22.06	70.59	
Seasons	Summer	30.77	16.67	12.18	59.62	0.229
	Winter	26.44	27.59	10.34	64.37	
	Rainy	21.60	21.60	17.60	60.80	

*M.synoviae=Mycoplasma synoviae*.

Table-2 shows that the seroprevalence of MS was highest (69%) in farm 5 among the different breeder farms under the study. The seroprevalence was found to be lowest (28%) in the farm 3. There was statistically significant (p<0.01) differences in the seroprevalence of MS with farm 3 to other breeder farms under study in Chittagong district.

The seroprevalence of MS infection in the breeder farms was highest (70%) with the flock size of >10,000, whereas it was lowest (57%) with the flock size ranging between 4000 and 7000 birds. From this result, it became evident that higher the flock size greater the seroprevalence of MS in the breeder farms of Chittagong district. There was a statistically significant (p<0.01) variation in terms of seroprevalence of MS among the different flocks of the breeder farms in Chittagong district (Table-2).

As shown in Table-2, the seroprevalence of MS in breeder farms of Chittagong district was lowest (42%) in the lowest age group (10-19 weeks) and highest (70%) in the highest age group (>60 weeks) of birds. This gives an indication that as the age of birds advanced the seroprevalence of MS infection in the breeder farms was also advanced. There was a statistically significant (p<0.01) difference in the seroprevalence of MS in the different age groups of birds from various breeder farms of Chittagong district.

The seroprevalence of MS in the winter season was found as high as 64%, whereas it was lowest (60%) in the summer season. However, the difference in the seroprevalence of MS was statistically not significant (p>0.05) for the winter, summer, and rainy seasons in the breeder farms of Chittagong district.

**Figure-1:**
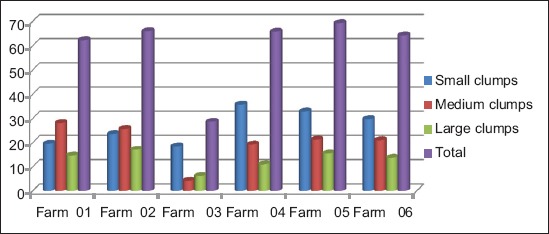
Strength of the agglutination reaction in (serum plate agglutination) test in different breeder farms.

The large clumps may be due to the presence of the highest concentration of antibody. Sera samples forming large clumps were also reacted faster than other small clump forming sera samples. As shown in Table-2 and Figure-1, the large and very large sized clumps in SPA test were found in the highest percentage (17.02%) in farm 2, and the medium sized clums were found highest (28.05%) in farm 1. The small sized clumps were found in the highest percentage (35.62%) in farm 4. It is to be mentioned also that in farm 3 the strength of agglutination reaction was lower than the remaining other farms.

The highest seroprevalencewas found 69.22% and lowest was 28.57% among the breeder farms under study in Chittagong district. The overall seroprevalence of MS was found 60%.

**Figure-2:**
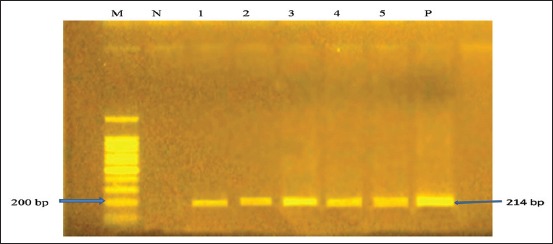
Results of polymerase chain reaction for 16S rRNA gene of *Mycoplasma synoviae*; Lane M: 100 bp ladder; Lane N: Negative control; Lane 1-5: 16S rRNA gene-sized (214 bp) amplicon; Lane P: Positive control.

The PCR product of MS for the respective primer (16S rRNA gene) was migrated as 214 bp band on agarose gel (Figure-2). In the PCR test, each of the farms under study showed a positive result. Therefore, it may be concluded that every seropositive farms under study were harboring MS infection.

## Discussion

In this study, only positive samples were used to analyze data because the all the seronegative samples were found negative in PCR, so we can assume that agglutination test is quite cheapest and easiest method than PCR in MS diagnosis. In this study, only 16S RNA reported primer was used for screening the MS and compares the test result with agglutination test primarily.

MScan be transmitted vertically and horizontally [2]. Results show that the highest (69%) and lowest (28%) prevalence of MS infection was found in farm 5 and 3, respectively. In farm 3, strict “culling of flocks” was practiced that probably reduced vertical transmission of *Mycoplasma* to the offspring resulting in lowest prevalence. This finding is in agreement with a similar study demonstrated by Feberwee *et al*. [27], who reported the lowest MS infection (6%) in farms where intensive culling was practiced. The high prevalence (69%) of MS infections in breeder stock can be explained by the frequent occurrence of multiple age housing and lower biosecurity standards in farm 5, and this argument again has been supported by the study of Stipkovits and Kempf [2] and Kleven *et al*. [28]. The higher prevalence of *Mycoplasma* infection might also be due to the replacement of breeding stock with the progeny of the same flock [8]. Intensive nature of poultry farming also affords opportunity for recycling of the pathogens due to population density [14] that might also explain why the breeder farms under study showed the higher prevalence of MS. The other factors that contribute MG infection are poor ventilation, contamination of litters and no restriction on the movement of the technical personnel, visitors, and such other persons as well as other biosecurity measures [29]. No relation was found between a special breed and occurrence of MS infection status [25,30]. Size of flock was found not to have much effect up to the flock size of 10,000 which conforms with the work of Seifi and Shirzad [25], who reported as saying that size of flock does not have an impressive effect in appearance of MS infection, but this problem is worse in the greater sizes of flocks. Our findings of large flocks having >10,000 birds showing seroprevalence of 70% and smaller sized flocks of bird (4000-7000) showing prevalence of 57% is in agreement with Heleili *et al*. [31] and Talha [32], who showed a higher infection rate (76.97%) in large flocks (18,000 birds) in comparison to small flocks (500-1000 birds) [25] showed the highest infection rate (57%) in large scale flocks (>40,000 birds) in comparison (41.9%) to small (up to 30,000 birds) flocks. Similar report was demonstrated by Catania *et al*. [30]. This variation may be due to horizontal transmission of infection, deficiency in management, and low biosecurity [8,14,27,29].

Age is a very important parameter influencing the incidence of mycoplasmosis [9,33,34]. The seroprevalence of MS was found higher in the advancing age groups. The highest (70%) and lowest (57%) prevalence of MS was found in age groups >60 weeks and 10-19 weeks, respectively. This finding was in agreement with other research groups. Feberwee *et al*. [27] reported that the seroprevalence of MS was highest (60%) in age group above 51 weeks. The same type of findings with 12% seroprevalence in age group 10-20 weeks and 43% seroprevalence in age group above 60 weeks was suggested by Seifi and Shirzad [25] and Mukhtar *et al*. [35]. The prevalence study of Yegani and Korver [36], which was based on the detection of MS antibodies in eggs, reported a prevalence of 78.6% in commercial layer flocks in East England. In another study of Qasem *et al*. and Hammouda *et al*. [37,38], 87% prevalence of MS was found in commercial layer flocks in Southern California. The infection was associated with older flocks that had been molted or frequently medicated. Stipkovits and Kempf, [2] Bonneaud *et al.*, [39] and David *et al*. [40] suggested that it may be due to more exposure of MS organism to birds as their age advances because MS can be transmitted both vertically and horizontally. In the present study, seasonal variation for the prevalence of MS was observed. However, the statistical analysis showed no significant (p>0.05) variation among the prevalence of seasons; the seroprevalence was highest (64%) in winter and lowest (60%) in summer (Table-2). This finding was in agreement with Heleili *et al.*, [9] Seifi and Shirzad [25], Hossain *et al.*, [41] and Sikder *et al*. [42] where they suggested that higher prevalence in winter might be due to the influence of cold weather. However, this finding was not in agreement with the findings of Heleili *et al*. [31] and Arbelot *et al*. [43] where they found that the seroprevalence was higher in summer season. In the present study, the overall seroprevalence of MS was (60%) which is in agreement with Giasuddin *et al* [11]. He reported the seroprevalence of MS in Bangladesh to be 61-67%.

For eradication of MS infection, rapid and accurate identification of MS is of great importance and molecular methods such as the PCR have been developed to improve this. Earlier MS-specific PCRs were based on the 16S rRNA gene [44,45], and more recently, some have been based on hemagglutinin genes [15,46]. In this study, species-specific primers of Pérez *et al*. [22] were used. These MS primers were selected from the 16S rRNA gene. The PCR method of Garcia *et al*. [44] used by Pourbakhsh *et al*. [24] is also based on the 16S rRNA gene. The PCR used in this study was species specific and had been used in recent years by other workers successfully [22,24]. In this study, all seropositive farms showed a positive result in PCR. With these findings, it may be concluded that those farms were harboring MS infection. It is possible to determine the presence of serum antibody using SPA test but not possible to determine whether the antibody is due to active infection or vaccination. To determine active infection or presence of MS, either organism culture or PCR test can be used. However, *Mycoplasma* being a facultative organism its culture is time-consuming and laborious [47]. On the other hand, PCR is comparatively easy, less time-consuming, and most reliable diagnostic tool. Therefore, PCR was adopted in this study for confirmation of the presence of MS in the infection [26,48].

These results strongly support the use of this PCR assay as an efficient alternative or supplement to culture and serological identification, which are labor-intensive, extremely time-consuming, and often provide confusing results. Overall, it is suggested that the PCR could be an alternative method for accurate identification of the MS infection, especially in breeder chicken flocks [49].

MS infection was found to be prevalent among the poultry breeder farms in Chittagong district. The overall seroprevalence of the disease was 60% with the highest and lowest rate of 69% and 28%, respectively. The prevalence varied significantly among the different farms due to varied farm conditions, age groups, and flock sizes; however, there was not any seasonal variation.

The prevalence rate of MS that has been observed in this study may not be true for the whole country. To determine the actual prevalence rate on MS in Bangladesh, a detail study covering the whole country may be conducted. Economic impact due to MS infection in poultry and the strain of MS involved was not assessed through this study. For proper control of the disease, strain identification of MS has a statistically significant value. Further study is needed to be conducted to have all those questions answered.

## Authors’ Contributions

MIC, KMK: Designed the research work and provided the technical guidance. MHA and MSI: Conducted the research work. SMZHC, ABS, and MNA: Provided necessary help during research. MIC and KMK: Helped in laboratory analysis. MNA and TMR: Revised the manuscript. All authors read and approved the final manuscript.
